# Inferior oncological prognosis of surgery without oral chemotherapy for stage III colon cancer in clinical settings

**DOI:** 10.1186/1477-7819-12-145

**Published:** 2014-05-10

**Authors:** Jo Tashiro, Shigeki Yamaguchi, Toshimasa Ishii, Asami Suzuki, Hiroka Kondo, Yohei Morita, Kiyoka Hara, Isamu Koyama

**Affiliations:** 1 Department of Gastroenterological Surgery, Saitama Medical University International Medical Center, Yamane, Hidaka-shi, Saitama 350-1298, Japan

**Keywords:** Oral adjuvant chemotherapy, Colorectal cancer, Relapse-free survival, Overall survival, Capecitabine, Tegafur plus leucovorin

## Abstract

**Background:**

Cancer patients not admissible for adjuvant chemotherapy are generally at high risk of considerably inferior prognosis. The aim of this retrospective study was to evaluate poorer survival without administration of oral adjuvant chemotherapy of stage III colon cancer patients in clinical settings.

**Methods:**

Between April 2007 and September 2011, 259 patients with stage III colon cancer who underwent curative surgery were retrospectively assigned to the adjuvant chemotherapy group of 171 patients (66%) and the surgery alone group of 88 patients. Oral fluorouracil (5-FU) derivatives used in adjuvant chemotherapy, such as oral uracil and tegafur plus leucovorin (UFT/LV) or capecitabine, were the most commonly used.

**Results:**

The 3-year relapse-free survival (RFS) rates were 74.9% for all cases, 58.3% for the surgery alone group, and 83.4% for the adjuvant chemotherapy group (*P* = 0.0001). The chemotherapy group was associated with a dramatic improvement in survival for stage IIIB (surgery alone 57.7% versus adjuvant chemotherapy 83.9%; *P* = 0.0001) and stage IIIC (surgery alone 18.2% versus adjuvant chemotherapy 57.3%; *P* = 0.006) patients. There was a significant difference in the overall recurrence rate between groups (surgery alone 35.2% versus adjuvant chemotherapy 18.1%; *P* = 0.002). Multivariate analysis identified adjuvant therapy as an independent predictive factor of reduced recurrence (hazard ratio (HR): 3.231; *P* = 0.004) and improved RFS (HR: 2.653; *P* = 0.001).

**Conclusion:**

In clinical settings, adjuvant therapy was the only significant prognostic factor of survival. Since many patients prefer not to receive chemotherapy, it is critical to inform stage III colon cancer patients that chemotherapy raises their chances of survival by three-fold compared with curative surgery alone.

## Background

Adjuvant chemotherapy after curative resection for stage III colon cancer patients has been widely recommended as a standard treatment to prolong disease-free survival (DFS) and overall survival (OS) since the early 1990s [1,2]. The National Surgical Adjuvant Breast and Bowel Project (NSABP) reported the results of a surgical adjuvant clinical trial (protocol C-03) that indicated significant extension of DSF and OS in stage II and III colon cancer patients who received fluorouracil (5-FU) plus leucovorin (LV) compared with patients who received semustine, vincristine, and 5-FU (protocol C-04) [3-5]. Furthermore, the NSABP protocol C-06 demonstrated that oral uracil and tegafur (UFT) plus LV (UFT/LV) were associated with DSF and OS rates similar to those obtained with a regimen of intravenous weekly bolus 5-FU plus LV (5-year DFS 67.0% versus 68.2%, 5-year OS 78.5% versus 78.7%) [6-8]. 5-FU significantly improves DFS and OS rates over surgery alone, with relative risk reductions of 30% and 26%, respectively [9].

The Xeloda® in Adjuvant Colon Cancer Therapy (X-ACT) trial was undertaken to compare the efficacy and tolerability of oral capecitabine with 5-FU/LV (intravenous bolus) as adjuvant therapy for patients with stage III colon cancer [10,11]. The 3-year relapse-free survival (RFS) rates were 65.5% with capecitabine and 61.9% with 5-FU/LV, for an absolute difference of 3.6% (95% CI, −0.9 to 8.1; *P* = 0.12). The 3-year OS rates were 81.3% with capecitabine and 77.6% with 5-FU/LV, giving an absolute difference of 3.7% (95% CI, −0.1 to 7.5; *P* = 0.05) between treatment groups. Since oral capecitabine offers equivalent clinical benefits in terms of efficacy, safety, convenience, and treatment cost, it can replace intravenous 5-FU/LV in the adjuvant treatment of stage III colon cancer.

Generally, patients not receiving adjuvant chemotherapy have considerably inferior prognosis. The Japanese Society for Cancer of the Colon and Rectum (JSCCR) reported recurrence rates of 30.8% for stage III colon cancer [12]. The present retrospective study evaluated the prognosis of stage III colon cancer patients excluded from adjuvant chemotherapy because they were older patients, had a high risk of severe comorbidities, or simply refused to receive treatment.

The limitations of the study are that it has retrospective clinical observations, a lack of patient randomization, and a dependence on the information available in the patient files. Which is more benefit or more harm associated with various treatment strategies is decided depending on the situation of each patients in clinical settings. Therefore, the aim of this study was to demonstrate the poor prognosis without oral adjuvant chemotherapy for stage III colon cancer patients in clinical settings.

## Patients and methods

### Patient selection criteria

Between April 2007 and September 2011, 259 patients with stage III colon cancer who underwent curative surgery were retrospectively assigned to the adjuvant chemotherapy group (n = 171) or the surgery alone group (n = 88). The database contained detailed information on patient characteristics, operative findings, histology, laboratory findings, and adjuvant therapies. The follow-up survival data were collected retrospectively through medical record analyses. The exclusion criteria were as follows: rectal cancer; malignancy other than colon cancer; and patients who did not receive adjuvant chemotherapy or did not have any information about the chemotherapy. Cancer was staged using the American Joint Committee on Cancer (AJCC) 7th edition TNM classification: colon and rectum [13].

### Chemotherapy adjuvant regimens

All patients started receiving adjuvant ≥8 weeks after curative surgery. They were required to have an Eastern Cooperative Oncology Group (ECOG) performance status of 0 to 2, to have signed informed consent, and be aged between 20 and 80 years old. The JSCCR recommends four adjuvant therapy regimens for stage III colorectal cancer: intravenous 5-FU/LV, UFT/LV, capecitabine, or FOLFOX (5-FU/LV plus oxaliplatin). In Japan, given the expected benefits and possible risks of toxicity, a consensus has not been reached as to whether adjuvant regimens containing oxaliplatin should be given to stage III patients. Several oral 5-FU derivatives are available, such as oral UFT/LV or capecitabine, and they are preferred for their convenience. Consequently, these were the most commonly used regimens in this study [14].

The UFT regimen is a preparation of tegafur-uracil in a molar ratio of 1:4. Tegafur is the 5-FU prodrug converted to 5-FU in the liver. LV is used to modulate 5-FU biochemically, and has been widely adopted for the treatment of advanced colorectal cancer [8,15]. The 5-week cycles of chemotherapy consisted of 4 weeks of oral UFT/LV, followed by 1 week of rest, and were repeated for ≥6 months. The UFT was administered at a dose of 300 mg/m^2^/day, and LV was administered at a dose of 75 mg/day. The daily doses of UFT/LV were divided into three doses administered every 8 h with water. Patients were instructed to avoid food from 1 h before to 1 h after each dose. Capecitabine is an oral fluoropyrimidine that generates 5-FU preferentially in tumor tissue through a three-step enzymatic cascade [16]. The adjuvant was administered at a dose of 1.250 mg/m^2^ twice a day for 14 days, followed by 7 days of rest. Standard care included a total of eight cycles. The amount of drug received was based on reported pill counts and patient declarations at the end of each cycle.

### Follow-up protocol

The patients were scheduled for follow-up visits every 3 months during the first 3 years, every 6 months during the next 2 years, and annually thereafter. Each visit included a physical examination and computed tomography (CT) scans of the chest, abdomen, and pelvis. Colonoscopies were performed during the first, third, and fifth year of follow-up. The median follow-up period was 41 months (range: 3 to 73 months).

### Data analysis

Statistical analysis was performed using SPSS version 21.0 software (SPSS Inc., Chicago, IL, USA). The chi-squared test or Fisher’s exact probability test were used to compare recurrence rates. Logistic regression analysis was conducted on the parameters found to be significantly associated with recurrence by chi-squared tests or Fisher’s exact probability test (*P* <0.05) to identify the independent factors of recurrence. Survival rates were calculated by the Kaplan–Meier method and compared by the log-rank test. Stepwise forward Cox regression model was conducted for parameters found to be significantly associated with survival by the log-rank test (*P* <0.05) in order to identify the independent factors of survival. Values of *P* <0.05 were considered significant in all analyses. This analysis was conducted using the intention-to-treat theory.

## Results

### Patient characteristics

The two groups were similar with respect to gender, tumor location, depth of invasion, tumor differentiation and lymphovascular invasion, and TNM sub-classification (IIIA/IIIB/IIIC) (Table 1). However, mean age was significantly higher in the surgery alone group (63.4 versus 75.4 years; *P* <0.0001). The causes for adjuvant chemotherapy rejection were older patients (>80 years), high risk of severe comorbidities or postoperative complication, and self-judgment of refusal.

**Table 1:** Comparison of patient characteristics stratified according to receipt of adjuvant chemotherapy and rejected reasons of adjuvant chemotherapy

**Variable**	**Surgery (n = 88)**	**Adjuvant chemotherapy (n = 171)**	***P *** **value**
Gender			0.78
Female	38	77	
Male	50	79	
Age (years, mean)	75.4	63.4	<0.0001
Comorbidity	60 (68%)	82 (47%)	0.002
ASA (grade ≥3)	27 (30%)	2 (1%)	<0.0001
Location			
Right side of colon	36	62	0.47
Left side of colon	52	109	
Colon	77	136	0.11
Rectosigmoid	11	35	
Serum CEA			
≥5	36 (41%)	73 (43%)	0.78
≥10	19 (22%)	43 (25%)	0.53
Postoperative hospital stay (mean)	10.8	8.9	0.08
Histological type			
Differentiated	79	156	0.7
Undifferentiated	9	15	
Lymphovascular invasion (≥2, 3)	26 (30%)	46 (27%)	0.65
Tumor depth			
T1/T2/T3/T4	13/5/52/18	9/15/113/34	0.06
Lymph node metastasis			
N1 (1 to 3)	65	131	0.33
N2 (4≤)	23	40	
Lymph node number (≥12)	81 (92%)	161 (94%)	0.52
TNM stage			
IIIA/IIIB/IIIC	16/60/11	22/127/22	0.380
Rejected reasons of adjuvant chemotherapy			
Older person (>80 years)	37 (42%)		
Self-judgment	29 (33%)		
Severe comorbidities	16 (18%)		
Postoperative complications	6 (7%)		

ASA, American Society of Anesthesiologists physical status classification system; CEA, carcinoembryonic antigen; TNM, tumor, node, metastasis.

### Prevalence of each adjuvant regimen

The UFT/LV regimen was performed in 104 patients (61%), capecitabine in 59 patients (34%), and other types in eight patients (5%; S1 and single-agent of UFT). The overall compliance was 77%, as 40 patients discontinued the therapy without evidence of a protocol event. The most common symptoms of drug toxicity were severe nausea and diarrhea (3.5%); the risks of severe hepatitis, neutropenia, and hand foot syndrome (5%) only with capecitabine. The completion rate of adjuvant chemotherapy was significantly less in patients with UFT/LV (72%) than with capecitabine (83%; *P* <0.0001).

### Survival analysis

**Figure 1:**
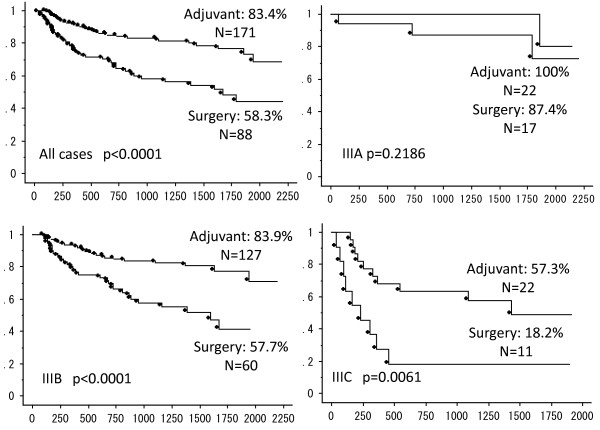
**Kaplan–Meier estimates of 3-year relapse-free survival (RFS) of all cases, and patients with stage IIIA, IIIB, and IIIC colon cancer.** Survival analysis compares the surgery alone group and chemotherapy group.

**Figure 2:**
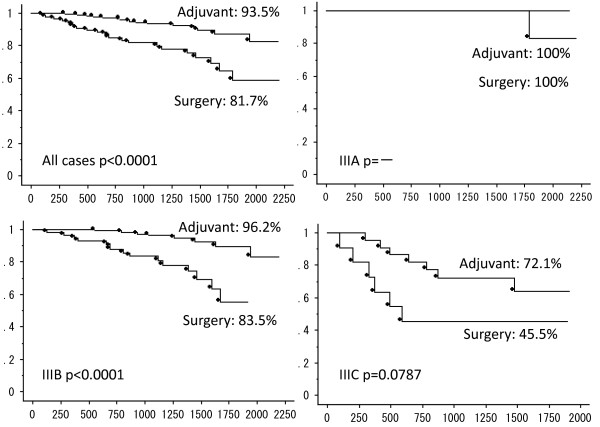
**Kaplan–Meier estimates of 3-year overall survival (OS) of all cases, and patients with stage IIIA, IIIB, and IIIC colon cancer.** Survival analysis compares the surgery alone group and chemotherapy group.

The Kaplan–Meier estimates of 3-year OS and 3-year RFS survival are presented in Figure 1 and Figure 2, respectively. When the two groups were compared, the adjuvant chemotherapy was associated with a significant improvement in 3-year OS (surgery alone: 81.7% versus adjuvant chemotherapy: 93.5%; *P* <0.001) and RFS (surgery alone: 58.3% versus adjuvant chemotherapy: 83.4%; *P* <0.001). Furthermore, chemotherapy did not affect the 3-year OS of stage IIIA and IIIC patients, and the 3-year RFS of stage IIIA patients.

Univariate and multivariate analyses were conducted for each clinicopathological factor (Table 2). Based on univariate analysis, advanced age was associated with poorer survival, whereas adjuvant chemotherapy was associated with improved survival. Multivariate analysis was performed to identify independent predictors of survival. Chemotherapy was the only significant prognostic factor of improved survival (hazard ratio (HR): 0.379; 95% confidence interval (CI), 0.214 to 0.670; *P* = 0.001), aside from oncological factors (depth of tumor invasion and TNM stage).

**Table 2:** Univariate and multivariate regression analysis for relapse-free survival (RFS) of stage III colon cancer

**Variable**	**Comparison**	**Univariate**	**Multivariate**		
		***P *** **value**	**HR**	**95% CI**	***P *** **value**
Gender	Female versus male	0.24			
Age		0.001	1.01	0.986 to 1.043	0.324
Comorbidity	No versus yes	0.745			
ASA (grade ≥3)	No versus yes	0.008	1.000	0.505 to 1.98	0.999
Location	Right versus left side of colon	0.306			
	Colon versus rectosigmoid	0.406			
Serum CEA	<5 versus ≥5	0.074	0.84	0.519 to 1.366	0.49
Operation	Laparoscopic versus open surgery	0.082	1.01	0.597 to 1.700	0.98
Tumor depth	T1, 2 versus T3, 4	0.002	0.26	0.089 to 0.733	0.01
Lymph node metastasis	N1 (1 to 3) versus N2 (4<)	0.004	0.71	0.387 to 1.301	0.27
TNM stage	IIIA, B versus IIIC	<0.0001	0.4	0.204 to 0.797	0.01
Adjuvant chemotherapy	No versus yes	<0.0001	2.65	1.48 to 4.756	0.001

ASA, American Society of Anesthesiologists physical status classification system; CEA, carcinoembryonic antigen; CI, confidence interval; HR, hazard ratio; TNM, tumor, node, metastasis.

Cancer recurrence occurred in 62 patients (recurrence rate (rec rate), 23.9%) during the follow-up period. The risk of recurrence during the first 3 years was two-fold higher for surgery alone (rec rate, 35%) than with adjuvant chemotherapy (rec rate, 18%). The overall median time to recurrence after initial resection for colon cancer was 10.5 months. In the surgery only group, the recurrence sites included the liver (12 patients, 38%), lung (five patients, 16%), dissemination (two patients, 6%), local site (three patients, 10%), and lymph node metastasis (four patients, 13%). In the adjuvant chemotherapy group, the recurrence sites were the liver (seven patients, 22.5%), lung (seven patients, 22.5%), dissemination (seven patients, 22.5%), local sites (one patient, 3%), and lymph node metastasis (seven patients, 22.5%).

Multivariate analysis was performed to identify independent predictive factors of recurrence (Table 3). Chemotherapy was the only significant predictive factor of recurrence (HR, 3.076; 95% CI, 1.422 to 6.653; *P* = 0.004) apart from oncological factors (depth of tumor invasion and TNM stage).

**Table 3:** Multivariate regression analysis for cancer recurrence of stage III colon cancer

**Variable**	**Comparison**	**Multivariate**		
		**HR**	**95% CI**	***P *** **value**
Age		1	0.968 to 1.036	0.941
Comorbidity	No versus yes	0.96	0.490 to 1.864	0.895
ASA (grade ≥3)	No versus yes	1.34	0.452 to 3.994	0.595
Location	Right versus left side of colon	1.08	0.561 to 2.089	0.81
Serum CEA	<5 versus ≥5	0.61	0.316 to 1.167	0.135
Operation	Laparoscopic versus open surgery	1.08	0.503 to 2.301	0.85
Morbidity	No versus yes	0.64	0.189 to 2.154	0.47
Tumor depth	T1, 2 versus T3, 4	0.17	0.037 to 0.801	0.03
Lymph node metastasis	N1 (1 to 3) versus N2 (4<)	0.56	0.244 to 1.268	0.16
TNM stage	IIIA, B versus IIIC	0.29	0.106 to 0.785	0.02
Adjuvant chemotherapy	No versus yes	3.23	1.458 to 7.159	0.004

CEA, carcinoembryonic antigen; CI, confidence interval; HR, hazard ratio.

## Discussion

Recently, practice-based research (studies based on medical care) which got rid of the distance of a clinician and the researcher tends to be regarded as important [17]. It is a valuable study method that provides information from daily clinical situations. The present study provides the survival outcome of stage III colon cancer patients. We report that patients treated only by curative surgery for various reasons had considerably inferior prognosis than those receiving both surgery and chemotherapy. Oncologists are aware of this, but tend to disregard factors affecting the efficacy of adjuvant chemotherapy, such as age, comorbidity, and postoperative patient conditions. Cohort studies including these parameters would provide a more comprehensive analysis of cancer treatment outcome. For patients intolerant to anticancer drugs, cancer recurrence and prognosis due to aggravation of serious comorbidity are important factors influencing survival. The survival rates we present for surgery alone are more realistic because they account for the patients who refused chemotherapy for economic, social, or philosophical reasons.

The 3-year RFS rate of the surgery alone group was significantly lower than for the chemotherapy group. In addition, chemotherapy was associated with a two-fold lower cancer recurrence rate during the first 3 years for stage III colorectal cancer patients, with 18% and 35%, respectively. Both stage IIIB and IIIC patients benefited from chemotherapy with a dramatic improvement in survival rate over surgery alone. Multivariate analysis only identified chemotherapy as an independent predictive factor for improvements in prognosis and reduction in cancer recurrence. Therefore, patients unable or unwilling to receive chemotherapy have a poor oncological prognosis. It is critical to provide this information to the patients unable to receive adjuvant chemotherapy for stage IIIB and IIIC. When recurrence occurs in stage IIIB and IIIC colon cancer patients, the oncologic surgeon who makes the decision of surgery alone treatment will provide the best supportive care.

The present study emphasizes the needless of chemotherapy in cases of stage IIIA colon cancer. Whereas both groups had an excellent outcome, chemotherapy after surgery raised the 3-year RFS from 87.4% to 100%. While the sample sizes were too small to reach statistical significance, it is likely that chemotherapy could be omitted for certain types of stage IIIA colon cancer with low risk of recurrence, such as node positive T1/T2 patients. Adjuvant administration must be decided based on the status of each patient with stage IIIA colon cancer. While the benefits of 100% 3-year RFS is important for patients, it must be weighed against the adverse effects.

We must determine how to improve the treatment of patients with poor performance status, severe comorbidities, and older patients who are not normally eligible for chemotherapy [18]. The older age patient group is commonly overlooked in clinical trials and, as a result, is often undertreated despite the proven efficacy of adjuvant therapy [19,20]. The X-ACT trial added to the growing volume of evidence suggesting that age alone should not be a barrier to the use of adjuvant chemotherapy in colon cancer [21,22]. On the other hand, performance status should be the governing factor. In addition, it is important to remember that renal impairment is more common in older patients, and is associated with an increased risk of toxicity with UFT/LV or capecitabine. Renal function should therefore be assessed before the onset of chemotherapy. The most appropriate type of chemotherapy requires a risk-benefit assessment of each adjuvant regimen available based on age, disease stage, performance status, comorbidities, and patient preference. For cancer patients hospitalized for severe comorbidities, an immunity activation agent (that is, polysaccharide-K) may be added to single-agent UFT, but the impact on recurrence has not been determined [23].

## Conclusion

Although other reports have documented treatment outcomes in stage III colon cancer, the present study analyzed a rare subset of colon cancer patients (stage IIIA, IIIB, and IIIC) treated only with oral adjuvant chemotherapy at a single institution. Receiving adjuvant chemotherapy was the only prognostic factor for an improved treatment outcome in a clinical setting. Patients that are uncertain about the benefits of adjuvant chemotherapy should be informed of the poor survival rates for stage IIIB and IIIC colon cancer, as well as the excellent survival rates for stage IIIA colon cancer without adjuvant chemotherapy. Unfortunately, stage IIIB and IIIC colon cancer patients who cannot undergo adjuvant chemotherapy because of severe comorbidities, an advanced age, or by choice, need to be informed of the inferior prognosis without treatment.

## Abbreviations

5-FU: Fluorouracil; AJCC: American Joint Committee on Cancer; ASA: American Society of Anesthesiologists physical status classification system; CEA: Carcinoembryonic antigen; CI: Confidence interval; CT: Computed tomography; DFS: Disease-free survival; ECOG: Eastern Cooperative Oncology Group; HR: Hazard ratio; JSCCR: Japanese Society for Cancer of the Colon and Rectum; LV: Leucovorin; NSABP: National Surgical Adjuvant Breast and Bowel Project; OS: Overall survival; rec rate: Recurrence rate; RFS: Relapse-free survival; TNM: Tumor, node, metastasis; UFT: Uracil and tegafur; X-ACT: Xeloda in Adjuvant Colon Cancer Therapy.

## Competing interests

The authors declare that they have no competing interests.

## Authors’ contributions

JT acted as guarantor of the integrity of the study, conceived the study concept, designed the study, defined the intellectual content, performed the literature research, undertook clinical and experimental studies, performed data acquisition, data analysis, and statistical analysis, and prepared and edited the manuscript. SY reviewed the manuscript. All authors read and approved the final manuscript.
